# Flexible Wearable Tri-notched UWB Antenna Printed with Silver Conductive Materials

**DOI:** 10.1021/acsomega.4c05071

**Published:** 2024-09-12

**Authors:** Wendong Yang, Xi Cheng, Xun Zhao, Jia Wang

**Affiliations:** † Institut für Physik, Institut für Chemie, Center for the Science of Materials Berlin, Humboldt-Universität zu Berlin, Berlin 12489, Germany; ‡ School of Electronic and Information Engineering, Liaoning Technical University, Huludao City 125105, China; § Helmholtz-Zentrum Berlin für Materialien und Energie GmbH, Berlin 14109, Germany

## Abstract

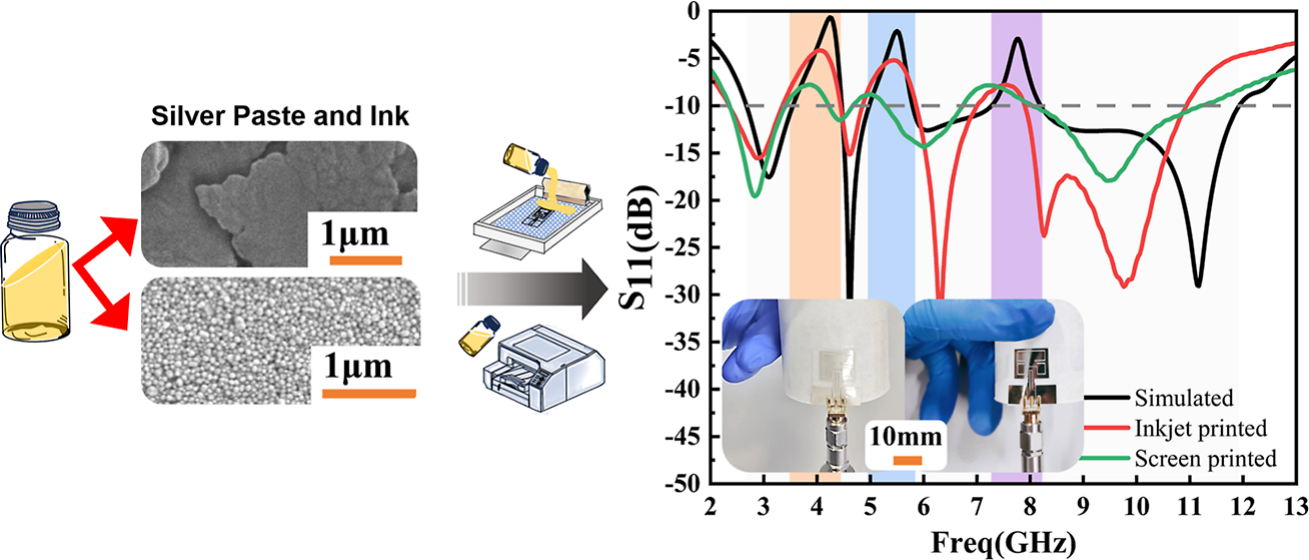

The advancement of Internet of Things and associated technologies has led to the widespread usage of smart wearable devices, greatly boosting the demand for flexible antennas, which are critical electromagnetic components in such devices. Additive manufacturing technologies provide a feasible solution for the creation of wearable and flexible antennas. However, performance reliability under deformation and radiation safety near the human body are two issues that need to be solved for such antennas. Currently, there are few reports on compact, flexible ultrawideband (UWB) antennas with more notch numbers, reliable bendability, and radiation safety. In this paper, a UWB antenna with trinotched characteristics for wearable applications was proposed and developed using printable conductive silver materials consisting of silver microflakes or silver nanoparticles. The antenna has a compact size of 18 × 20 × 0.12 mm^3^ and adopts a gradient feeder and a radiation patch with three folding slots. It was fabricated on transparent and flexible poly(ethylene terephthalate) film substrates, using screen printing and inkjet printing. The measurement results demonstrated that the fabricated antennas could cover the UWB band (2.35–10.93 GHz) while efficiently filtering out interferences from the C-band downlink satellite system (3.43–4.21 GHz), wireless local area networks (4.66–5.29 GHz), and X-band uplink satellite system (6.73–8.02 GHz), which was consistent with the simulation results. The bendability and radiation safety of the antennas were evaluated, proving their feasibility for usage under bending conditions and near the human body. Additionally, it was found that the screen-printed antenna performed better after bending. The research is expected to provide guidance on designing flexible antennas that are both safe to wear and easily conformable.

## 1 Introduction

With the development of the Internet of Things and 5G technologies, personal mobile and smart wearable devices are becoming increasingly popular.^1^ Flexible antennas are extremely significant in the field of wearable electronics,^2^ especially in biomedical monitoring^3^ and wireless communication application.^4^ Ultrawideband (UWB) technology can provide short-range, high-bandwidth communications at very low power levels and is a good candidate for wearable devices.^5^ However, some narrowband systems, such as wireless local area networks (WLAN) system and C-band downlink satellite system, may cause interference to the UWB systems.^6^ Consequently, it is imperative to effectively filter these interferences in order to enhance the communication quality of UWB systems. Recently, UWB antennas with multinotch properties that are miniaturized, low-profile, flexible, and easy to integrate have become a popular subject;^7,8^ however, the majority of UWB antennas with notch characteristics are designed on rigid substrates like FR4,^9^ Rogers,^10^ and so on, which are difficult to be integrated on wearable devices. Additive manufacturing technologies, such as inkjet printing, screen printing, and three-dimensional (3D) printing, have been used for the fabrication of flexible antennas due to their convenience and the progress in the synthesis of the materials.^11,12^

So far, the developed flexible antennas can be roughly divided into two groups based on the substrate type: antennas made of textile substrates^13,14^ and antennas made of flexible polymers^15−17^ or paper substrates.^18^ Both types of antennas employ conductive materials to create the conductive part of the antenna using different printing techniques. Compared with other printing techniques,^19−21^ inkjet printing offers the advantages of a simple operating principle, fewer manufacturing steps, and real-time design and verification, which has lately been widely used in the fabrication of flexible antennas.^22,23^ However, inkjet printing has higher requirements for the fluid properties of the ink to ensure good printing quality. Screen printing is a mature printing technique that creates a pattern by forcing a conductive paste via a screen. Although it belongs to plate printing that cannot be revised in real-time, it has the advantages of low cost, strong ink adaptability and easy printing of thick films.^24^

In terms of antenna design, the combination of UWB technology and flexible antennas is a research hotspot.^25^ Flexible UWB antennas can fulfill the demands of wireless technology for wide bandwidth and fast transmission rates while also being flexible and wearable. Currently, there are numerous reports on this topic. In 2019, Ramos-Silva et al.^16^ created a flexible UWB antenna on a polyamide (PI) substrate with a dimension of 40 × 55 mm^2^, which operates in the frequency range of 2–20 GHz. In 2020, Hasan et al.^18^ reported an inkjet-printed bendable circular monopole based on polyethylene terephthalate (PET) photographic paper, with a dimension of 34 × 25 × 0.135 mm^3^ and an operating bandwidth of 1.66–56.1 GHz. In 2021, Kirtania et al.^22^ designed an inkjet-printed bendable UWB antenna on a PET substrate with dimensions of 47 × 25 × 0.135 mm^3^, which operates at 3.04–10.70 and 15.18–18 GHz (Ku upper band). In 2023, Ibrahim et al.^26^ proposed a chemically etched flexible broadband antenna. The antenna is made on a flexible substrate RO3003, with dimensions of 38 × 41 × 0.254 mm^3^, and operates in the 2.4–10 GHz. In the UWB frequency bands, signal interferences from some narrowband bands will worsen the communication quality; thus, researchers have invented notch techniques. These techniques focused on designing specific structures so that the antenna exhibits significant reflections or attenuation in a specific frequency band to inhibit signal transmission in that band. The common methods include etching slot on the radiation patch, integration of parasitic branches, fractal technique, using multilayer dielectric structures and electromagnetic band gap (EBG) structures, and so on.^8^ Lakrit et al.^27^ presented a flexible UWB antenna with notch properties for WLAN applications, which was fabricated on Teflon substrate. The antenna has dimensions of 42.5 × 30 × 0.6 mm^3^ and operates at a frequency of 3.25–13 GHz. A split ring resonator (SRR)-based stop band filter embedded in the ground plane was adopted to mitigate the interference from WLAN band. Zou and Jiang^28^ presented a fractal UWB antenna with band notch characteristic. The antenna, printed on a PI substrate, has an impedance bandwidth of 15.48 GHz (3.6–19.08 GHz) and a size of 20.5 × 13.9 × 0.125 mm^3^. By introduction of two symmetrical complementary SRRs (CSRRs) on the ground plane, the band rejection of the X-band uplink frequency from 7.9 to 8.41 GHz was achieved. Geyikoglu et al.^29^ proposed a dual-notch UWB antenna fabricated on the PI substrate. The antenna has dimensions of 60 × 62 × 0.125 mm^3^ and operates within a bandwidth of 2.05–14 GHz. Notch characteristics at the WiMAX and HyperLAN/2 bands are achieved by adding two triangular helical slots of different sizes to the defected ground structure. Compared with other notch techniques such as etching various slot on the ground plane incorporation of electric ring resonator (ERR), SRRs or CSRRs, using parasitic elements, and EBG resonators, introducing slots with different shape on the radiation patch is simple in structure and easy to operation, requiring just the opening of a gap (slot) in the antenna’s metal conductive part. By adjustment of parameters such as the length and width of the slot, the desired notch properties can be achieved.

Although progress has been made in developing flexible UWB antennas with notch features, significant constraints remain. First, flexible UWB antennas with triple-notch characteristics and a small size have rarely been reported. The present trinotched flexible UWB antennas are relatively large in size, with lengths and widths greater than 20 mm, making them difficult to integrate into smaller communication devices. Second, these antennas do not consider radiation safety while operating close to the human body, especially in wearable device applications. Finally, the majority of these antennas are fabricated using inkjet printing, with only a few based on screen printing. Although there have been studies evaluating the radiation safety of antennas^23,30^ or investigating the effects of different printing technologies on printed antenna performence,^31,32^ there are few research on flexible notch UWB antennas of small size that consider both bending performance and the radiation safety, as well as the impact of printing technologies.

This paper proposes a compact, flexible UWB antenna with trinotched characteristics. The antenna has dimensions of 18 × 20 × 0.12 mm^3^ and is designed on a transparent, flexible, and low-cost PET substrate. The notch characteristics were achieved through the use of a gradient feedline, a radiation patch with three folding slots and a coplanar waveguide (CPW) feed structure. Screen and inkjet printing were employed to fabricate the antenna prototypes using silver conductive materials. Prototype tests showed that the operating bandwidth of the antenna covers the targeted UWB frequency band and achieves the desired notch properties. Bending tests in different directions and curvature radii, as well as specific absorption rate (SAR) tests, revealed that the antenna has great radiation performance and good bendability, and fulfills the wearability requirements for humans. The flexible, bendable, compact size, low profile, and low SAR value of the proposed antenna make it suitable for use in wearable devices.

## 2 Materials and Methods

### 2.1 Antenna Design

The proposed flexible triple-notch antenna is designed to operate in the UWB band (2.35–10.93 GHz), which can shield interferences in the C-band downlink satellite system (3.43–4.21 GHz), WLAN (4.66–5.29 GHz) and X-band uplink satellite system (6.73–8.02 GHz). Flexible and transparent PET film, was chosen as the substrate for the designed antenna. Its dielectric constant, tangent loss, and thickness are 4, 0.01, and 0.12 mm, respectively.

**Figure 1:**
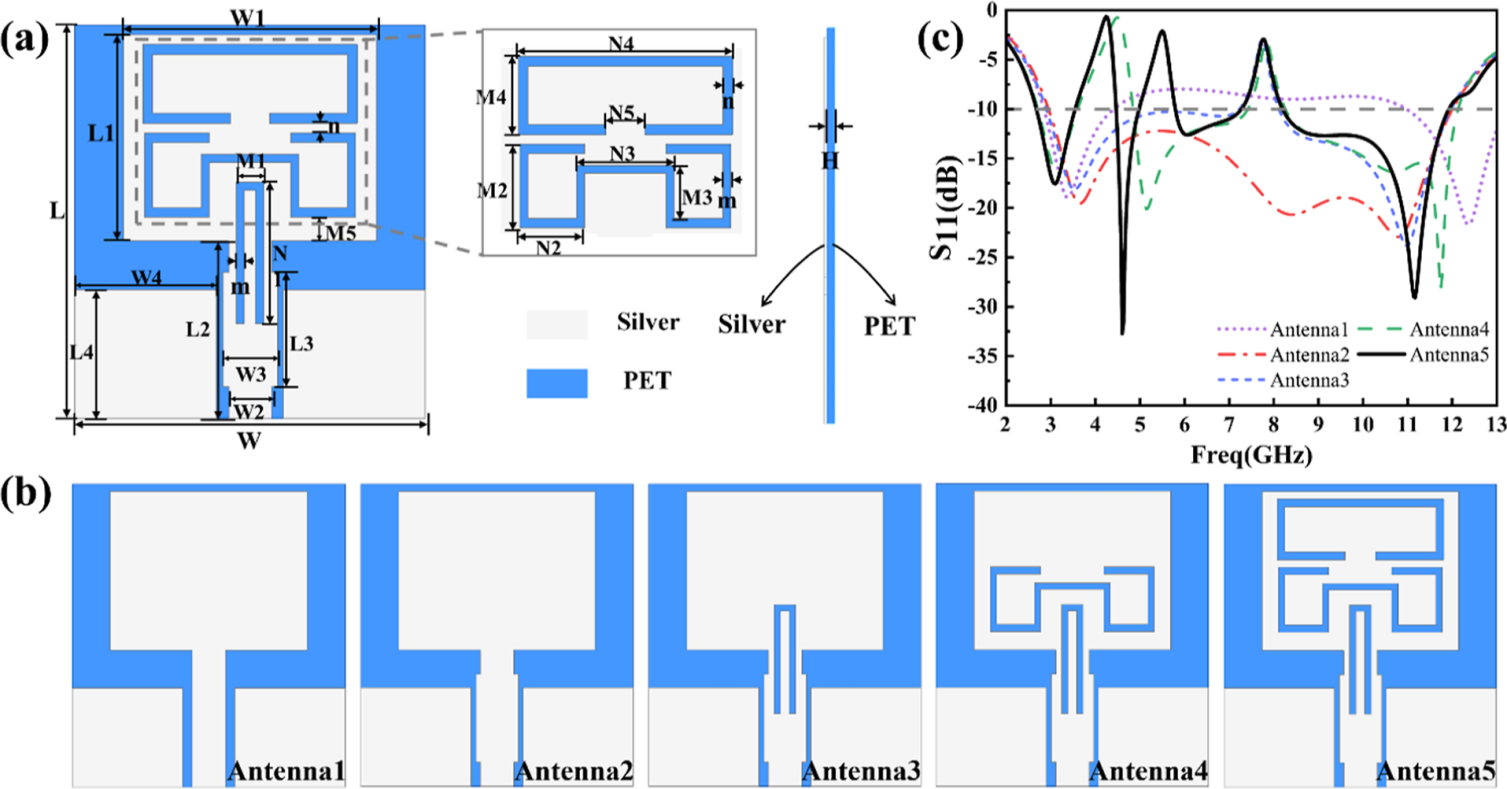
(a) Schematic view of the proposed antenna, (b) structural evolution processes, and (c) the corresponding reflection coefficient S_11._.

Figure 1 shows the final structure, design evolution procedures, and associated reflection coefficients (S_11_) at each design step of the proposed antenna. The white part represents the silver conductive layer, which is made of screen-printed silver paste or inkjet-printed silver nanoink, while the blue part is the flexible PET substrate. Figure 1a displays the shape and dimensions of the proposed tri-notched UWB antenna. The antenna was made of a CPW feeder (matched to the 50 Ω feedline) and a rectangular patch with three folding slots. Its dimensions are defined by the variables *W*, *L*, *H*, *W*1, and *L*1, which correspond to the substrate width, substrate length, substrate thickness, patch width, and patch length, respectively. These parameter values are calculated using eqs 1–6.
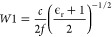
1
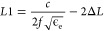
2
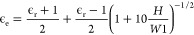
3
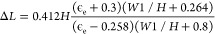
4
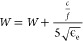
5
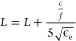
6where *c* is the speed of light, *f* is the center frequency of the antenna’s bandwidth, ϵ_r_ is the dielectric constant of the substrate; ϵ_e_ is the effective permittivity, which can be calculated by eq 3, and Δ*L* is the length of the equivalent radiation gap, which can be calculated by eq 4.

Based on the equations shown above, the initial values for *W*, *L*, *W*1 and *L*1 are calculated to be 13.5, 10.7, 17.9, and 15.0 mm, respectively. After optimization, these four parameters are determined to be 13, 10.5, 20, and 18 mm, respectively.

Figure 1b shows the design process of the proposed antenna. The UWB and notch characteristics were achieved by adopting a gradient feedline structure (Antenna 2) and introducing three folding slots (Antennas 3–5) on the radiation patch, respectively. Feedlines with gradient shapes can provide wider bandwidth.^33^ As shown in antennas 1 and 2 (Figure 1b,c), the bandwidth of the antenna was extended from 2.86–4.39 to 2.67–11.93 GHz, covering the UWB band. The main mechanism for bandwidth broadening of this gradient feedline is through improved impedance matching and electromagnetic field distribution. Additionally, antennas with such feed structure can achieve lower return loss over a wider frequency range.

Slotting technology is adopted to realize the notch properties of the antenna, because it is simple to perform. The size and shape of the slot can be calculated according to the formula of the notch frequency function.^8^ In this paper, the length of the slot is determined by using eq 7.
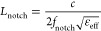
7
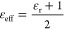
8where *c* is the speed of light, *f*_notch_ is the center frequency of the notch, and ε_eff_ is the effective permittivity, which can be calculated by eq 8, where ε_r_ is the dielectric constant of the substrate.

In combination of the center frequencies (3.95, 5.475, and 7.5 GHz) of the three notch bands and eq 1, the lengths of the slots were determined to be 24.0, 17.3, and 11.6 mm, respectively. Based on these values, the ultimate lengths of the three slots after optimization were 27.8, 24.0, and 14.2 mm. From Figure 1b,c, it can be clearly observed that the addition of these slots successively realized the desired notch characteristics to the X-band uplink satellite system (7.25–7.75 GHz), C-band downlink satellite system (3.7–4.2 GHz), and WLAN band (5.125–5.825 GHz).

### 2.2 Antenna Fabrication

**Figure 2:**
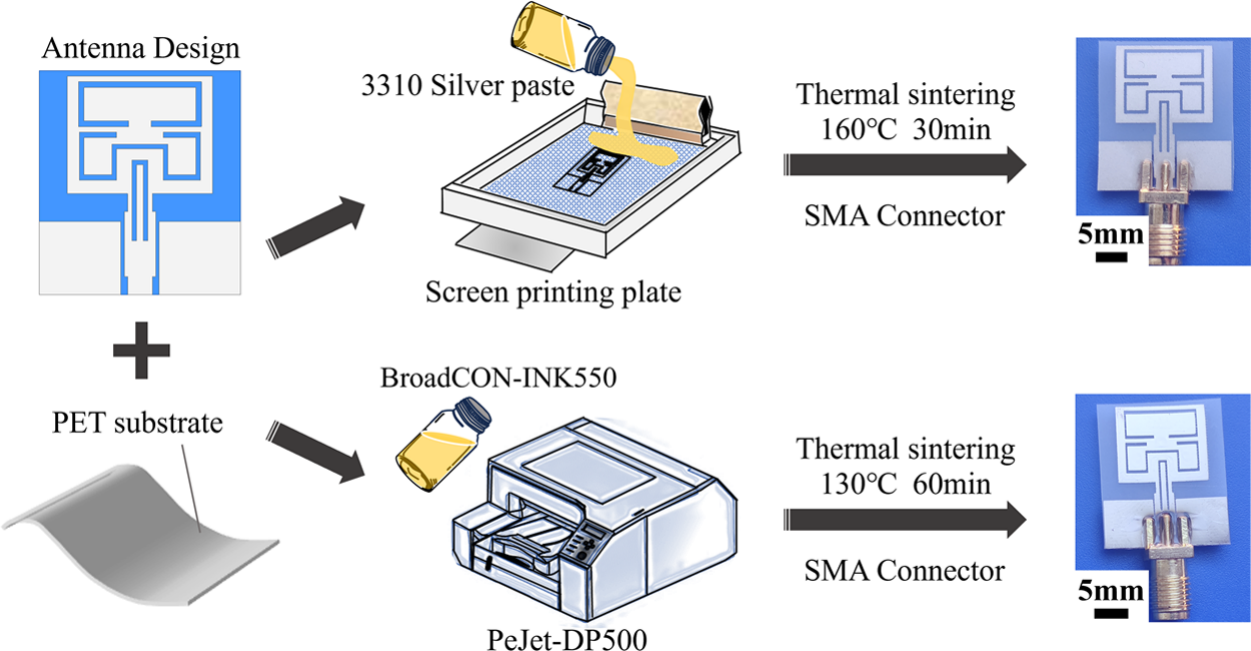
Fabrication processes of the proposed antenna using screen and inkjet printing.

At present, inkjet printing is favored in the fabrication of flexible antennas. However, the advantages of screen printing, such as low cost and easy to print,^34^ cannot be ignored. In addition, few studies have examined the effects of different printing methods on the performance of the obtained antennas. Therefore, in this paper, the antenna was fabricated by using both screen printing and inkjet printing to examine the differences between them. Figure 2 shows the fabrication process of the proposed antenna.

The flexible PET substrate and the optimized antenna pattern are first prepared. Then, the antenna pattern was screen-printed using a conductive silver paste (3310, Delo Co., Ltd., China) on the PET substrate and was also inkjet-printed with a silver nanoparticle ink (Ink 550, BroadTeko Co., Ltd., China) for comparison. Finally, the printed antenna patterns are sintered (160 °C, 30 min for screen printing; 130 °C, 60 min for inkjet printing) to evaporate organic solvents to obtain the required electrical properties. The antenna prototype was created by connecting the sintered antenna pattern with an SMA (Sub Miniature version A) connector using conductive glue.

### 2.3 Characterization

The thermal decomposition behaviors of silver paste were explored using a simultaneous thermal analyzer (DSC-TG, HITACHI STA200), with a heating rate of 10 °C/min under N_2_ atmosphere. A field-emission scanning electron microscope (SEM, TESCAN MIRA LMS) and a surface energy disperse spectrometer (EDS, Oxford Xplore30) were used to inspect the surface, cross-section morphology and chemical composition of the printed films. The resistivity was calculated from the sheet resistance measured by a four-point probe system and the thickness of the film. The S_11_ values of the antenna prototype were determined using a vector network analyzer (Keysight E5063A).

## 3 Results and Discussion

### 3.1 Performance of Printable Silver Paste

Conductive pastes, as an important material in the electronic industry, have been used in light-emitting diode,^35−37^ liquid crystal display,^38,39^ integrated circuit chips,^40^ and other electronic devices^41,42^ because of their outstanding performances. Such paste is usually composed of a conductive phase, bonding phase, solvent, and other additives. The conductive phase offers the conductive pathway, the bonding phase provides the basic mechanical properties, and the solvent gives the paste essential fluid properties for screen printing. Metal silver costs less than platinum and gold and has higher conductivity, stability, and oxidation resistance than carbon or copper. Thus, silver fillers such as silver particles, flakes, and nanowires are commonly utilized as typical conductive phases, and their morphology and structure have a significant impact on the properties of the paste. A commercial silver paste was chosen here and characterized thermally, electrically, and mechanically by DSC-TG, SEM/EDS and four-probe analyses to determine the important properties for the antenna applications. For inkjet printing, a silver ink was adopted, and its properties were reported in our prior work^43^ and they will not be described in detail here.

**Figure 3:**
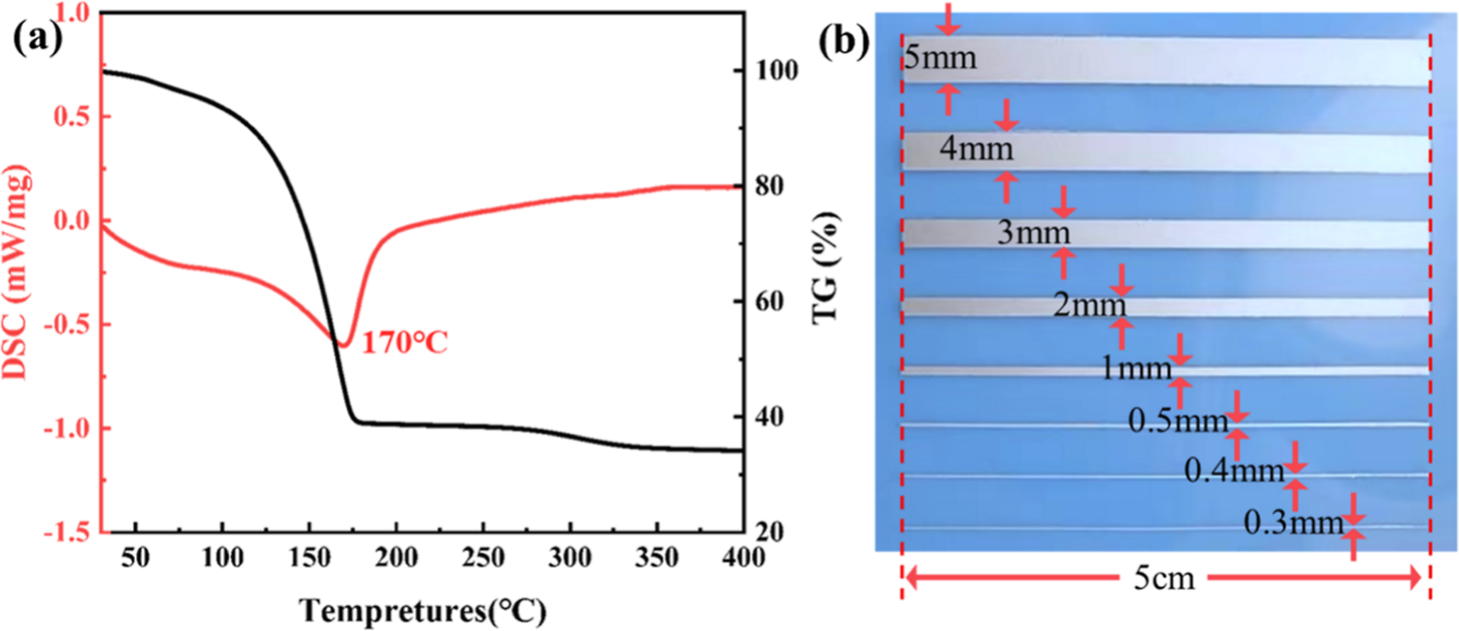
(a) DSC–TG results of the commercial silver paste and (b) printed lines of different widths.

The TG curve in Figure 3a demonstrates that the weight loss of the silver paste stabilizes at 170 °C, with a solid content of about 40 wt %. The DSC curve displays an obvious absorption peak around 170 °C, which might be from evaporation of the solvents and coalescence of the silver particles. Line width is one of the important indicators to evaluate the print quality. The smaller the width of the printed line, the higher the resolution. Here, lines of different widths were printed on PET substrates to assess the printability of the silver paste. As shown in Figure 3b, the printed lines are uniform and regular, with clear edges, and the minimum line width is 0.3 mm, which is consistent with the designed value, indicating that the silver paste adopted has good printability and can ensure the precision of the printed pattern in dimension.

**Figure 4:**
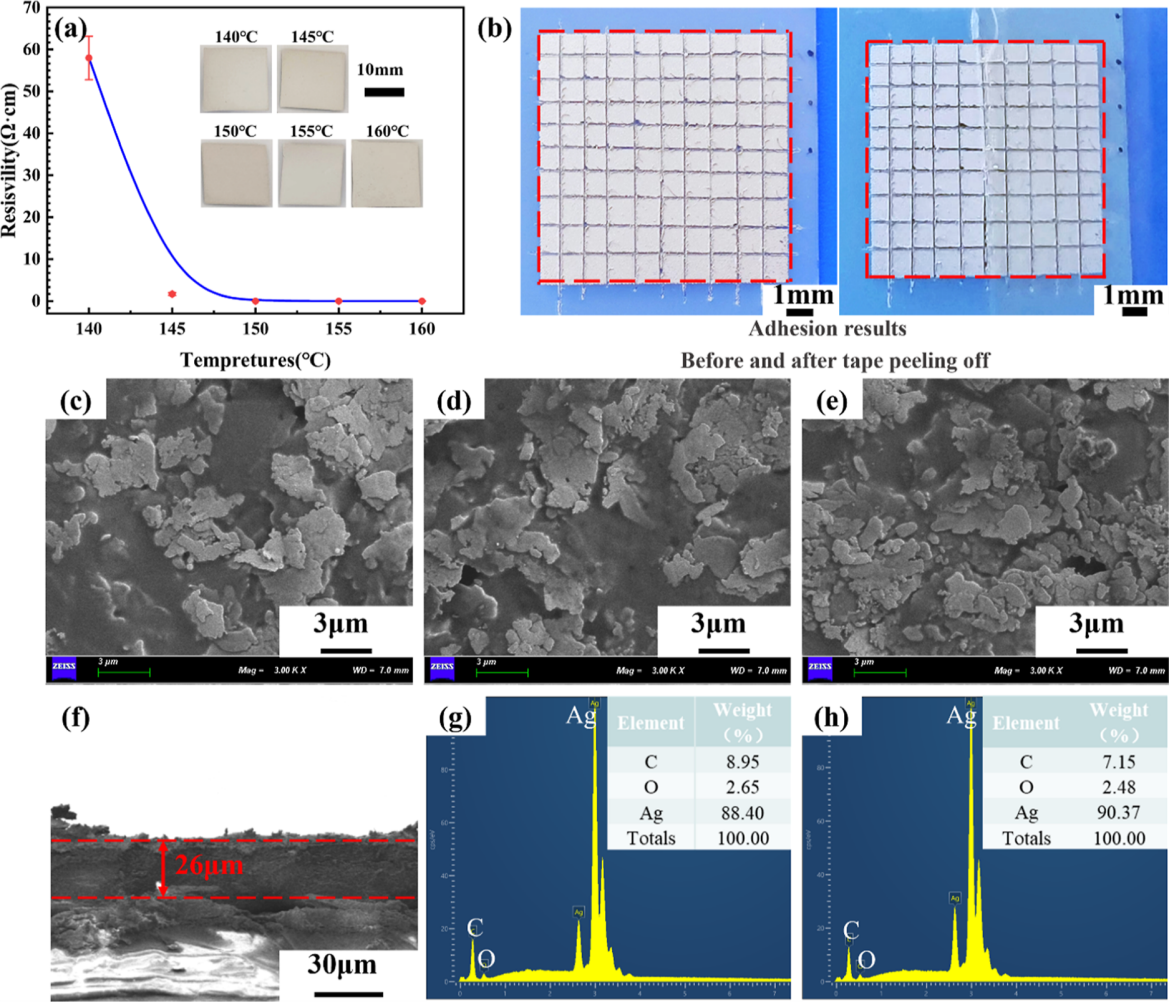
(a) The relationship between temperature and resistivity of the printed silver films, (b) adhesion measurement results; surface and cross-section microstructures as well as chemical composition of the printed silver films obtained at (c,g) 140, (d) 150, and (e,f,h) 160 °C.

Based on the results of thermal analyses and the heat resistance of PET substrates, 140–160 °C were chosen for the sintering of the screen-printed silver films to examine the relationship between temperature and resistivity. The resistivity of the printed films drops from 57.986 to 0.012 Ω·cm when the sintering temperature increases from 140 to 160 °C, as shown in Figure 4a. As for the reason, it is related to the evaporation or decomposition of organic solvents and binders in the silver paste at high temperatures. Generally, the electrical properties of the produced silver film are associated with three factors: the degree of organic residue in the paste, the particle size, and the particle contact area. At 140–145 °C, the majority of the organic matter in the silver paste decomposes and volatilizes, and the contact area between conductive particles increases, so the resistivity drops significantly. At 145–160 °C, the particles produced have formed good connections and there is only a small amount of organic residue, therefore the resistivity changes slowly. In addition, the printed films exhibit homogeneous electrical properties since the resistivity values measured are almost same at different surface positions. The adhesion strength of the printed film on PET substrate was evaluated using a tape test according to the ASTM D3924 procedure. As shown in Figure 4b, the printed film exhibited a good adhesion of 5B, which is beneficial for antenna application.

Figure 4c–e shows the surface morphology of the silver films at temperatures of 140, 150, and 160 °C, respectively. The silver in the paste exhibits a flaky morphology with sizes in a micron range. Besides, some colloidal substances cover the surface of these silver flakes, and their amounts gradually decrease as the temperature increases. At high temperatures (160 °C), more silver flakes are exposed and have better contact with one other. The cross-section of the film prepared at 160 °C is shown in Figure 4f. It can be observed the printed silver conductive layer has a thickness of about 26 μm. There are some separations between the silver layer and the substrate that are caused by the cutting procedure used to create the cross-sectional sample.

Figure 4g,h depicts the chemical composition of the printed silver films. Each film was found to contain three main elements: carbon (C), oxygen (O), and silver (Ag). With the increase of the temperature from 140 to 160 °C, the Ag content increased from 88.40 to 90.37 wt %, whereas the C content decreased from 8.95 to 7.15 wt %, indicating that the solvents in the paste were mostly removed. This is consistent with the results of Figure 4b–e, where the increase in sintering temperature caused evaporation/decomposition of the solvents in the silver paste, resulting in enhanced electrical properties.

Considering the limited thermal stability of PET and the thermal behaviors of the silver paste, 160 °C was finally chosen for antenna fabrication. Here, it should be noted that the sintering temperature of the silver paste affects the structural stability and electrical properties of the printed antenna pattern, which, in turn, affects the resonance point of the antenna. A low sintering temperature will increase the resistivity of the conductive part of the antenna, shifting the antenna’s resonance point to lower frequencies.^32^ Therefore, one must—consider this factor when evaluating the performance of the screen-printed antenna prototype.

### 3.2 Optimization of Antenna Parameters

An antenna’s structural parameters must be optimized to realize the design requirements. The sizes and distances of the radiating elements, such as the length (L3) of the feedline, the width (W4) of the CPW structure, the opening width (N5) of top “*U*”-like slot, and the distance (*n*) between the two folding slots at the top of the patch, are key parameters of the proposed antenna since they can affect the radiation characteristics, impedance matching, and frequency coverage of the antenna. Thus, these parameters are independently optimized before fabricating the antenna prototype. As shown in Figure S1 in the Supporting Information, the influences of the length L3 of the feedline and the distance *n* between the two folding slots at the top of the patch on the UWB and the notch performance of the antenna was taken as an example. The influences of the distance *n* on the resonance frequency of the antenna when keeping other parameters constant were concluded. An optimized value of 0.5 mm for *n* was eventually determined.

The dimension of the antenna is determined to be 18 × 20 × 0.12 mm^3^ after optimization, with detailed structural parameter values given in Table S1 in the Supporting Information.

**Figure 5:**
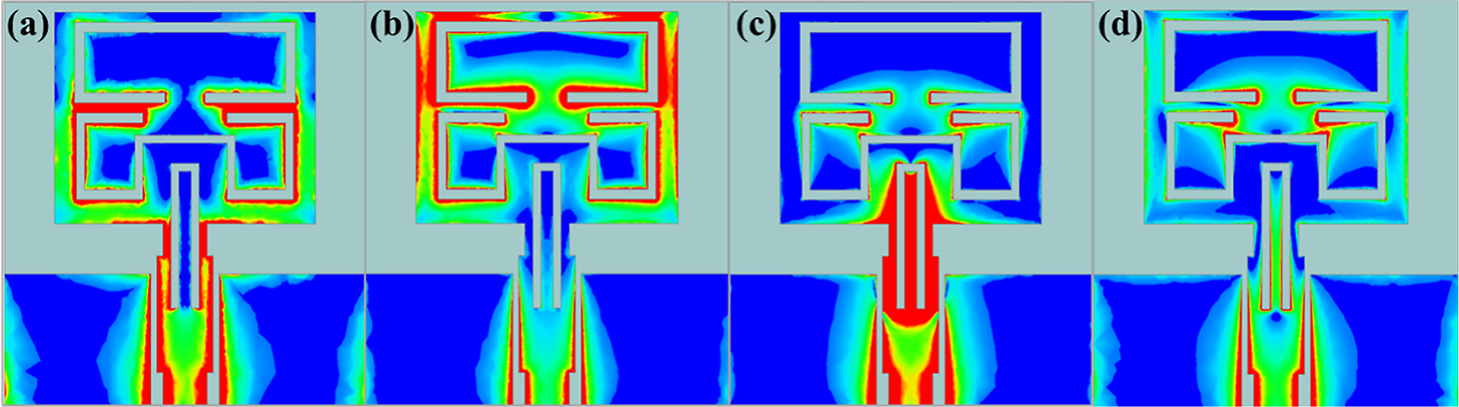
Surface current distribution at (a)3.5; (b)5.8, (c) 7.7 and (d) 9.0 GHz of the optimized antenna.

Figure 5 displays the surface current distribution of the optimized antenna structure at various frequencies. The colors in the figure depict the density of the surface current distribution with red being the greatest and pale blue representing the lowest. Figure 5a–c displays the surface current distribution of the antenna at notch center frequencies of 4.0 5.4, and 7.7 GHz. When the antenna operates at these frequencies, its surface currents are densely distributed near the slots where the notch is produced. At the higher UWB frequency of 9.0 GHz, as shown in Figure 5d, the surface currents are more evenly distributed on the antenna structure. This is consistent with the notch principle realized by slotting technology. The slot generates a high current density near it and a reverse current on both sides, resulting in the cancellation of the electric field. This reduces electromagnetic wave reflection in the relevant band, resulting in the expected notch characteristics.

### 3.3 Simulated Antenna Performance

**Figure 6:**
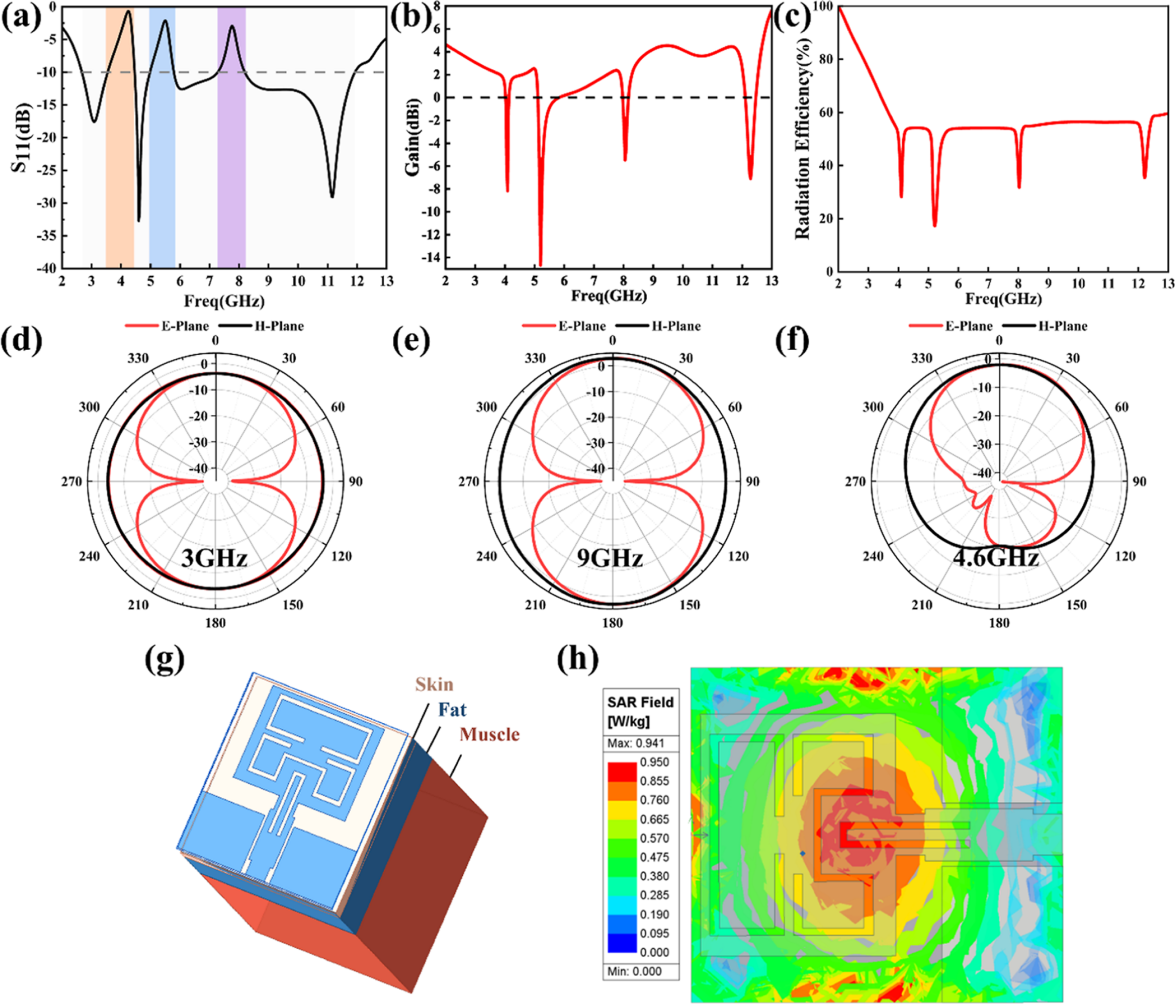
Simulated results of the proposed antenna (a) reflection coefficient S_11_, (b) gain, (c) radiation efficiency, (d,e) radiation pattern at 3, 9, and 4.6 GHz, (g)human tissue model utilized for antenna simulation, (h) SAR distribution of human tissue at 4.6 GHz, (i,j).

This section presents the performance of the optimized antenna. As shown in Figure 6a, the antenna operates in the range 2.67–11.93 GHz, completely covering the required UWB band and being even broader, making it suitable for use in UWB systems.

Meanwhile, the antenna achieves the notch characteristics in the frequency bands of 3.55–4.46, 4.98–5.79, and 7.28–8.20 GHz, which can filter out the interferences from the C-band downlink satellite system, WLAN, and X-band uplink satellite system to the UWB system. As shown in Figure 6b, the peak gain of the antenna varies from 2 to 4 dBi with an in-band average of 3.5 dBi in the UWB operating band. In the notch-band, the peak gain varies from −6 to 0 dBi with an in-band average of −4 dBi. The peak gain fluctuates very little within the UWB and notch bands, indicating that the radiation performance of the antenna remains stable and can ensure effective wireless signal transmission. The radiation efficiency in Figure 6c is stable around 57%, which indicates that the antenna has good radiation performance and can meet the requirements of UWB communication systems.

In addition to simulating the basic antenna properties, the interaction between the human body and antenna was also investigated. The parameter SAR was used to evaluate the antenna security performance for human body application. A human tissue model composed of skin, fat and muscle was established in HFSS software to simulate the effect of the antenna on human tissue (Figure 6g). The required tissue parameters were obtained from the literature,^23^ as shown in Table 1.

**Table 1:** Human Tissue Model Parameter at 4.6 GHz

body tissue	relative dielectric constant	dielectric loss angle	magnetic conductivity (s/m)	thickness (mm)
skin dry	36.099	0.29864	2.7588	2
fat	5.067	0.168	0.21784	8
muscle	50.058	0.2823	3.6162	23

During the simulation process, the antenna was placed at a height of 3 mm above the tissue, and the received power of the reference antenna was set to 0.1 W. Considering the feasibility, representative frequency points (resonance points and edge frequencies) were selected only for SAR value testing, with the results presented in Table 2. In Europe and China, a SAR value of 2.0 W/kg per 10 g of tissue is the upper limit for the human body to absorb electromagnetic waves. Obviously, the SAR values of all test frequency points are below than 2 W/kg. Besides, the SAR value of the antenna basically shows a trend of increasing as the frequency increases, especially in the surface tissues, which may be related to the attenuation of high-frequency electromagnetic waves in tissues and the skin effect. Moreover, due to the rapidly changing dielectric properties of the tissue, the SAR value of the antenna at the high-frequency boundary frequency is lower than the SAR value at other nearby frequencies. Since the simulation results at each frequency are similar, only the SAR values of the antenna at 4.6 GHz (the lowest resonant frequency-are given as an example; see Figure 6h. The simulated result shows that the peak SAR of the antenna at 4.6 GHz is 0.941 W/kg, which is within the safety limit, verifying the safety of the antenna for wearable applications.

**Table 2:** Simulated SAR_10g_ Values (W/kg)

frequency (GHz)	2.68	3.13	4.60	6.07	11.14	11.92
SAR	0.877	0.876	0.941	1.386	1.388	1.054

Figure 6d–f shows the radiation patterns of the antenna at the low frequency of 3 GHz and the high frequency of 9 GHz, as well as the radiation pattern on the human body model when the antenna works at 4.6 GHz. The antenna exhibits similar radiation characteristics to the dipole antenna when operating at 3 and 9 GHz. It has omnidirectional radiation characteristics on the *H* plane and is “8” shaped and directional on the *E*-plane. When the human body model is loaded, the back lobe of the antenna’s *E*-plane radiation pattern is significantly decreased. This occurs due to the absorption and reflection of electromagnetic waves by human body tissues, which attenuate the radiation intensity of the antenna close to the human body. Overall, the simulation results for both SAR value and radiation pattern show that the antenna’s impact on human health is within safe and acceptable range.

**Figure 7:**
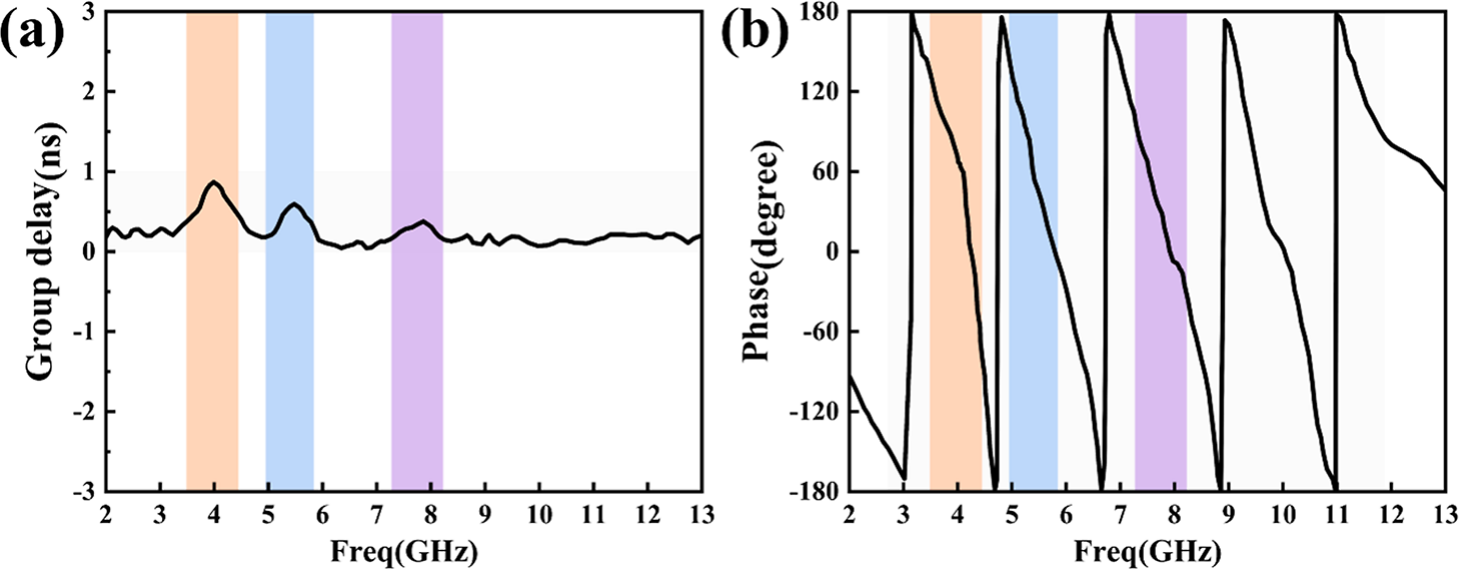
Simulated results of the proposed antenna in the time domain (a) group delay and (b) phase response.

In addition to frequency domain performance, time domain performance is also crucial for antennas. Here, the time domain performance of the antenna was analyzed using CST simulation software, and two important time domain parameters, group delay and phase, were examined. Figure 7a shows the antenna’s group delay characteristics throughout the whole frequency range. It can be observed that within the passband of 2–13 GHz, the antenna’s group delay is less than 1 ns, while inside the operational frequency band, the group delay is between 0 and 0.2 ns. These results show that the delay change of the signal as it passes through the antenna is small, indicating that the antenna has low distortion characteristics in a wide frequency band. Figure 7b shows the phase response curve of the antenna. As can be seen, in most frequency bands, the phase response curve of the antenna exhibits a decent linear change as the frequency increases. In the notch frequency bands (marked area), the group delay increases significantly, with fluctuations reaching up to 0.8 ns, and the phase response exhibits nonlinear changes. These changes imply that efficient signal suppression occurred in these bands; in other words, desirable notch characteristics were obtained. Overall, the antenna performs well in terms of group delay and phase response in the frequency range of 2–13 GHz, with low distortion and good reliability, making it suitable for multinotch UWB applications.

### 3.4 Antenna Conformal Performance

In addition to the stable and safe radiation characteristics, a flexible antenna is also expected to have a certain bendability for wearable applications. Therefore, the bending performance of the designed antenna is examined. Here, 7 GHz was chosen because it is the center frequency of the UWB frequency band and close to the third notch frequency band, allowing for a representative assessment of the antenna performance. In addition, this selection enables the test results to be used to evaluate the antenna’s entire performance under bending, including the notch characteristics before and after bending.

**Figure 8:**
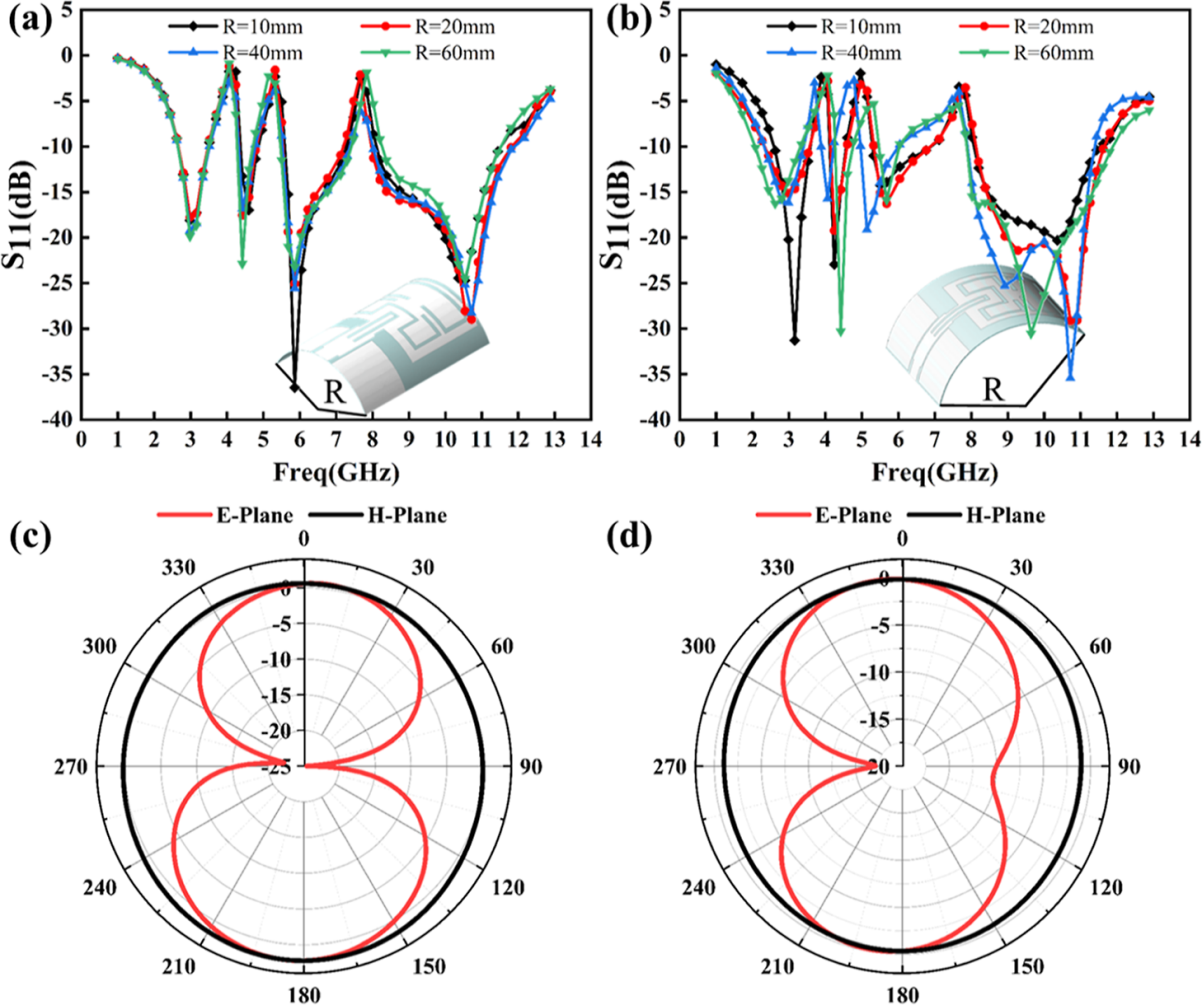
Reflection coefficient (S_11_) of the antenna when bent along the (a) *Y*-axis and (b) *X*-axis at a specified radius of curvature, and the radiation pattern at 7 GHz when bent along the (c) *Y*-axis and (d) *X*-axis with a curvature radius of 10 mm.

Figure 8 displays the S_11_ results of the antenna bending along the *Y-* and *X*-axes at bending radius (*R*) of 10, 20, 40, and 60 mm, and the radiation pattern of the antenna at 7 GHz and a bending radius of 10 mm. As shown in Figure 8a, when the antenna is bent along the *Y*-axis with different degrees, its operating frequency range and the notch center frequency are basically the same, showing performance similar to that without bending. When the antenna is bent to different degrees along the *X*-axis (Figure 8b), its operating frequency range changes slightly and the notch center frequency at low-frequency shifts, but it still exhibits tri-notched UWB characteristics. The change in S_11_ after bending is caused by a change occurring in the electrical length of the radiation structure. This is especially true when the antenna is bent along the *X*-axis since the surface current and radiation field distribution of the antenna change dramatically.

The radiation patterns of the antenna after bending are shown in Figure 8c,d. They remain unchanged when bent along the *Y*-axis but become distorted in the *E*-plane when bent along the *X*-axis. As for the reason, it might be that after bending along the *X*-axis, the antenna radiation structure changes, altering its impedance matching characteristics, allowing more current to be trapped inside the antenna, thereby leading to changes in the radiation pattern. In addition, two nulls can be observed in the *E*-plane pattern (Figure 8d) after the antenna is bent along the *X*-axis (long axis). The presence of these two nulls is primarily related to two factors: the distance between radiating elements and the surface current distribution of the antenna. As the antenna bends along the *X*-axis, significant physical deformation occurs, causing the distance between the radiating elements to change. This change causes destructive interference in specific directions. In addition, when the antenna is bent along the *X*-axis, the surface current distribution also changes, introducing phase differences between different parts of the antenna and causing the radiated fields to cancel each other in certain directions. The combination of these two factors results in the appearance of two nulls. Overall, the nulls in Figure 8d are associated with the changes in the physical deformation and current distribution generated by bending along the *X*-axis. This is acceptable for a bendable flexible antenna. The S11 results after the antenna is bent also confirm its suitability for bending conditions.

### 3.5 Measured Antenna Performance

**Figure 9:**
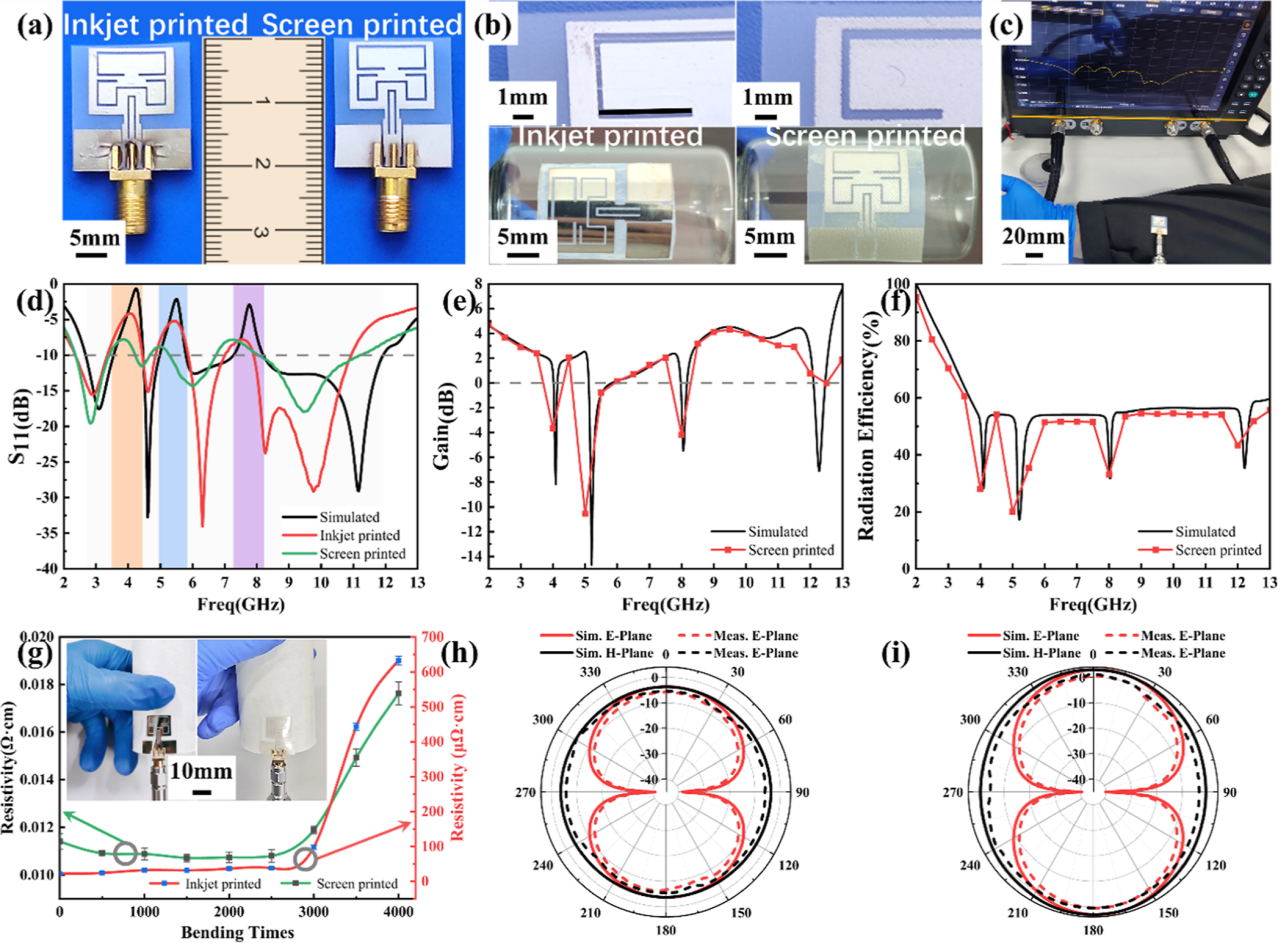
(a) Antenna prototype, (b) details of the printed antenna, (c) the measurement of antenna on arm, (d) reflection coefficient (S_11_) of the antenna prototype, (e) gain, (f) radiation efficiency, (g) the resistivity of the antenna after bending, and (h,i) radiation pattern at 3, 9 GHz.

Finally, two antenna prototypes were fabricated on flexible PET substrates using screen printing and inkjet printing, respectively, for performance verification. Figure 9a shows the optical photos of the fabricated two antenna prototypes, and Figure 9b displays the surface details of the two antennas. Obviously, there are some differences in their surface morphology. For screen-printed antenna, it has a rougher surface and a thicker conductive layer, while inkjet-printed antenna shows a smoother surface and a thinner conductive layer. Besides, the screen-printed antenna has a flatter surface than the antenna fabricated by inkjet printing. These differences are associated with the properties of the silver paste and ink utilized, which have an impact on the performance of the printed antennas. Reference (35) has demonstrated that the differences in printing technologies and materials will cause the reflection coefficient S_11_ curve of the antenna prototype to be frequency-biased. Both antennas also exhibit excellent bendability when attached to a cylindrical bottle with a radius of curvature of 7.5 mm (Figure 9b).

Given that the electrical performance of the antenna pattern after 160 °C thermal sintering is 4 orders of magnitude lower than that of an ideal conductor, we performed a secondary plasma treatment (300 W, 30 min) on the screen-printed antenna pattern, which yielded satisfactory results.

Figure 9d gives the measured S_11_ curves of the two antenna prototypes. As expected, both antennas showed the tri-notched UWB characteristics in the C-band downlink satellite system and WLAN and X-band uplink satellite systems, which are basically consistent with the simulation results. The resonance point of screen-printed antennas exhibits a minor low-frequency shift, possibly due to the properties of the silver paste, which results in a relatively high resistivity and rough surface morphology at 160 °C. The research in ref (32) indicated that the resonant frequency of the printed antenna was associated with the curing temperature of the ink as it impacts the electrical performance of the antenna pattern. At a low curing temperature, the resonant frequency of the antenna will shift to a low frequency due to the presence of the solvent of the ink (low conductivity). In our case, at 160 °C, the printed antenna pattern has a resistivity of 1.2 × 10^–2^ Ω·cm, which is 4 orders of magnitude lower than the resistivity of an ideal silver conductor (1.59 × 10^–6^ Ω·cm). This is why the resonance point of the screen-printed antenna shifts to a low frequency. Increasing the sintering temperature or adopting an alternative post-treatment method could improve the conductive properties of the silver film, enabling the resonance characteristics of the antenna to be closer to the ideal state. In addition, the measurement errors and losses caused by the SMA connection also have an impact on the measured results.

The near-body performance of the antenna was verified by an arm test. As shown in Figure 9c, it shows a stable performance. The radiation performance of the antenna in terms of patterns and gain was measured in an anechoic chamber. As shown in Figure 9e,f, the antenna has relatively low gain and efficiency at the frequencies of the three notch bands, which are in good agreement with the simulated results. Figure 9h,i illustrates the measured radiation patterns of the proposed antenna at 3 and 9 GHz, respectively. The radiation pattern of the *E*-plane is bidirectional, similar to that of the dipole antenna, while the radiation pattern of the *H* plane pattern is omnidirectional. These results are also consistent with the simulation results. Overall, the measurement results verify the rationality of the antenna in the design.

The electrical performance of antennas from the two printing techniques after multiple bending and deformations has also been investigated, as depicted in Figure 9g. The resistivity of both antennas becomes high after being bent 4000 times. When the number of bends is less than 2000, the resistivity of both the samples changes slightly. It also found here that the resistivity changes of screen-printed antenna are more stable after multiple bends than the one from inkjet printing, which may be related to the properties of the paste. For wearable flexible antennas, the stability of resistivity represents the bending resistance of the metal layer and the stability of antenna performance. Obviously, screen-printed antennas will have higher durability.

Table 3 compares the performance characteristics of the proposed antenna to those of other similar antennas in the literature. On the whole, the proposed antenna has the following three characteristics. First, it has a compact size, low profile, and simple structure, making it easy to be integrated on flexible and wearable devices. Second, it can filter out interferences from triple narrowband bands in the UWB system while having good bendability and safe radiation characteristics to human body. Lastly, compared to inkjet-printed antenna, the screen-printed antenna using silver micron-sheet paste has better bending resistance. These advantages make the proposed antenna a good candidate for applications in wearable devices.

**Table 3:** Comparison of the Proposed and Previous Antennas^a^

ref	dimensions (mm^3^)	bandwidth (GHz)	notch number	notch technique	bending test & direction		SAR test	fabrication approach	substrate
(15)	35 × 40 × 0.3	1.45	0		yes	two	no	inkjet printed	PET
(16)	40 × 55	2–20	0		no		no		PA
(17)	32 × 32 × 0.29	3.96	0		no		no	3D printed	PEN
(18)	34 × 25 × 0.135	1.66–56.1	0		yes	two	no	inkjet printed	PET paper
(22)	47 × 25 × 0.135	3.04–10.70, 15.18–18.0	0		yes	single	no	inkjet printed	PET
(26)	38 × 41 × 0.254	2.4–10	0		yes	single	no	chemical etched	RO3003
(27)	42.5 × 30 × 0.6	3.25–13	1	SRR	yes	two	no		teflon
(28)	20.5 × 13.9 × 0.125	3.6–19.08	1	CSRRs	yes	single	no		PI
(29)	60 × 62 × 0.125	2.05–14	2	slots	yes	two	no	airbrush printed	PI
(30)	60.3 × 60.3 × 3.5	2.40–2.48	0		yes	single	yes		PI
(43)	27 × 38 × 0.12	1.9–10.75	3	slots	yes	two	no	inkjet printed	PET
(44)	20 × 30 × 1	2.75–9.84	2	fractal	no		no		FR4
(45)	18 × 14 × 1	3.1–11.2	2	slots	no		no		FR4
(46)	17.6 × 16 × 0.12	2.9–10.61	3	slots	yes	single	yes	inkjet printed	PET
this work	18 × 20 × 0.12	2.35–10.93	3	slots	yes	two	yes	screen & inkjet printed	PET

a Note: PI, polyimide; PET, polyethylene terephthalate; PEN, polyethylene naphtholate; SRR, split ring resonator; CSRRs, complementary SRRs.

## 4 Conclusions

A wearable and bendable UWB antenna with trinotched properties was proposed based on printable conductive silver materials. The antenna has a small size (18 × 20 × 0.12 mm^3^), low profile, and fed by a CPW structure. The design addresses the integration problem that traditional microstrip antennas face in wearable devices due to their size and thickness. The antennas could be fabricated using screen printing or inkjet printing. The measurements showed that the antenna prototype created using both printing technologies covers the desired UWB band (2.35–10.93 GHz) and filters out the interferences from the C-band downlink satellite system (3.43–4.21 GHz), WLAN (4.66–5.29 GHz) and X-band uplink satellite system (6.73–8.02 GHz). The bending performance and radiation safety of the antenna were evaluated through bending and SAR tests, respectively. The results confirmed the reliability of the proposed flexible antenna for wearable applications. Furthermore, it was discovered that the screen-printed antenna performed well after bending. The proposed antenna has a compact dimension, which is also safe, reliable and has excellent anti-interference capabilities, making it a good candidate for usage in wearable devices.

## Supporting Information Available

Optimization of antenna parameters; dimensions of the optimized antenna (PDF)
