# Supplementary material for: Flexible Wearable Tri-notched UWB Antenna Printed with Silver Conductive Materials
Source: ACS Omega. 2024 Sep 12;9(38):39792–803. doi: 10.1021/acsomega.4c05071 (PMC11425645; doi:10.1021/acsomega.4c05071)
Supplement: Supplementary file 1 — ao4c05071_si_001.pdf [file ao4c05071_si_001.pdf]

Supporting Information

Flexible Wearable Tri-notched UWB Antenna Printed with Silver Conductive Materials

Wendong Yang[a, b, c*], Xi Cheng[b], Xun Zhao[b] and Jia Wang[b]

*a. Institut für Physik, Institut für Chemie, Center for the Science of Materials Berlin, Humboldt-Universität zu Berlin, Berlin 12489, Germany. Email: yangwendong@lntu.edu.cn; *corresponding author*

b. School of Electronic and Information Engineering, Liaoning Technical University, Huludao City, 125105, China.

c. Helmholtz-Zentrum Berlin für Materialien und Energie GmbH, Berlin 14109, Germany.

Section 1. Optimization of Antenna Parameters

Here, the influences of the length L3 of the feedline and the distance n between the two folding slots at the top of the patch on the UWB and notch performance of the antenna were taken as an example. When other parameters remain constant, changes in L3 can affect the impedance-matching state of the antenna, as seen in **Figure S1a**. When the length of L3 is 0 and 9.0 mm and the feedline is in a non-gradient state, the antenna has an impedance mismatch, making it impossible to cover the UWB band at low frequencies. After optimization, the antenna achieves satisfactory impedance matching when the L3 length is 5.8 mm, and can fully cover the entire UWB band.

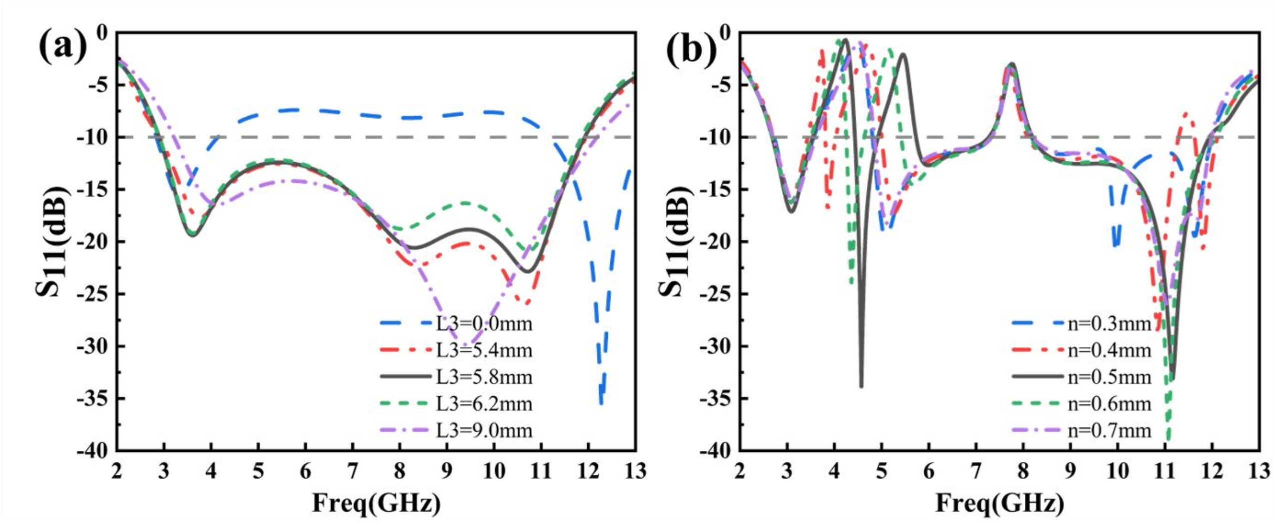

Figure S1. Influences of parameter values on the S_{11} of the proposed antenna: (a) L3 and (b) n

Section 2. Dimensions of the Optimized Antenna

Table S1. Parameters of the proposed antenna (Units: mm)

Parameter	Value	Parameter	Value	Parameter	Value	Parameter	Value
W	18	L1	10.5	N3	5	M4	4
L	20	L2	9	N4	11	M5	1.2
W1	13	L3	5.8	N5	2	H	0.12
W2	2.2	L4	6.3	M1	1.4	n	0.5
W3	2.8	N1	7.2	M2	4.3	m	0.4
W4	7.3	N2	3.3	M3	2.7		
